# Online and Social Media Data As an Imperfect Continuous Panel Survey

**DOI:** 10.1371/journal.pone.0145406

**Published:** 2016-01-05

**Authors:** Fernando Diaz, Michael Gamon, Jake M. Hofman, Emre Kıcıman, David Rothschild

**Affiliations:** 1 Microsoft Research, New York, NY, United States of America; 2 Microsoft Research, Redmond, WA, United States of America; Institut Pluridisciplinaire Hubert Curien, FRANCE

## Abstract

There is a large body of research on utilizing online activity as a survey of political opinion to predict real world election outcomes. There is considerably less work, however, on using this data to understand topic-specific interest and opinion amongst the general population and specific demographic subgroups, as currently measured by relatively expensive surveys. Here we investigate this possibility by studying a full census of all Twitter activity during the 2012 election cycle along with the comprehensive search history of a large panel of Internet users during the same period, highlighting the challenges in interpreting online and social media activity as the results of a survey. As noted in existing work, the online population is a non-representative sample of the offline world (e.g., the U.S. voting population). We extend this work to show how demographic skew and user participation is non-stationary and difficult to predict over time. In addition, the nature of user contributions varies substantially around important events. Furthermore, we note subtle problems in mapping what people are sharing or consuming online to specific sentiment or opinion measures around a particular topic. We provide a framework, built around considering this data as an imperfect continuous panel survey, for addressing these issues so that meaningful insight about public interest and opinion can be reliably extracted from online and social media data.

## Introduction

Online and social media provide an increasingly popular forum for public discussion of a large number of topics, including political conversations. Digital records of these discussions complement traditional approaches to opinion polling and offer the opportunity to better understand societal opinions at large [1]. Compared to traditional approaches, these sources have the advantage of *scale*. With billions of active participants globally, online and social media potentially capture the revealed actions and stated thoughts of a large segment of the population. These sources also have the advantage of *low latency*. Real-time online and social media data allow for continuous analysis as events unfold, and temporally-granular post-event analysis critical to isolating the impact of key sub-events.

In recent years, much research has focused on understanding the expression of political opinion online, and exploring its use as an alternative data collection modality for surveys and fundamental election data as a way to predict elections, identify support, and related tasks. The results are mixed. For example, while many papers have reported positive correlations between measures of social media and political outcomes [2,3,4], others have criticized their methodologies and reported contradictory, negative results [5,6,7]. There are two key reasons for potential problems in this research. First, it frequently focuses on election outcomes where standard data and methods are extremely accurate already. Second, it does not account for the uniquely non-stationary nature of the data as technology and its users evolve and, thus, how the data correlates with outcomes such as support, likeliness to vote, and donations. One clearly defined route for academic research is to improve upon standard objective outcome variables and it is reasonable to assume that this new data can improve these metrics. However, given these contradictory results it is worth exploring both new techniques and new outcome variables that may be more appropriate for this new data.

In this paper, rather than attempt to reproduce a traditional task, such as predicting election results from online and social media data, we instead take a step back and ask how this data differs from the traditional survey data:

*If we were to assume that online and social media data is the output of some hypothetical pseudo-survey methodology*, *how would this methodology differ from conventional survey techniques?*

We believe that a rigorous answer to this question–highlighting the fundamental differences between online and social media data and gold standard survey methodologies–can provide a new roadmap toward the use of such data. If earlier papers focus on treating the data like survey data, this paper defines the bounds of that assumption. To this end, we analyze what, to our knowledge, is the largest such corpora used for this purpose: the comprehensive search activity of a large panel of web users and the complete set of tweets during the period of the 2012 election cycle. We focus our analysis on two fundamental characteristics of a (pseudo-) survey methodology: (1) the dynamics of demographics and participation in the pseudo-survey; and (2) the dynamics of topics addressed in the pseudo-survey.

Our results extend beyond past research by illustrating how both participation and topical coverage are more dynamic and less predictable than traditional survey methodologies. In addition to demonstrating that participants in online and social media platforms are unrepresentative of the offline population, as characterized by key demographics such as gender and geography, our study shows that participation also shifts dramatically on a daily and even hourly basis, especially around key events. In other words, at the times when online and social media data’s real-time nature holds the most promise for providing timely insights, the composition of the participant base is most dramatically in flux.

As a result, we find that online and social media activity function like an opt-in panel where different users engage to different degrees during different times. Most existing research counts each of these engagements independently, ignoring user identity information. If this were a survey, it would be the equivalent of allowing users to respond as many times as they want. We explore the value of treating the online population as a panel rather than a cross-section, as there is a small fraction—but large number—of users who repeatedly discuss the same topics and dominate conversation.

Studying the topics addressed by participants in our hypothetical pseudo-survey, we find that the population of people searching and tweeting contributes different types of information at different times. Specifically, the topics they discuss shift during major events, as activity moves from content sharing to active commentary. If this were a survey, it would be equivalent to users answering systematically different questions at different times.

In short, if online and social media data are to treated as surveys, they must be treated as imperfect surveys indeed. Traditional surveys follow a rigorous procedure, asking the same question—Gallup has asked the same presidential approval question to its respondents since the late 1930s—to repeated cross sections of a random sample of a representative group of people. A search and social “survey,” however, is essentially polling a varying, non-random sample of voluntary participants who selectively respond to questions of their choice.

Despite their rigor, traditional surveys suffer from four well-known errors: sample, coverage, non-response, and survey design/execution; by considering online and social media in this context, we can better understand its potential valuable. Collectively this is called the total survey error, as defined in-depth in the literature [8,9]. Sample error is a result of only sampling a portion of the population, coverage error is the inability to reach the full population, non-response error is some people in the population not answering surveys if asked, and survey error is all of the error that comes from the design/execution of the survey. Online and social media data has a huge sample and its coverage is also strong for most populations, despite missing members of the general population without Internet access. While traditional surveys have responses rates below 10% and falling for election surveys [10], the equivalent response rate for online and social media data is much lower, as only a small fraction of a percent of the population online selects to discuss any topic on any given day. Additionally, the survey error is much worse because the researcher interpreting the data cannot control the questions, ordering, etc. Thus, even considering realistic estimates of total survey error for traditional surveys, they are likely to have a smaller error on the questions surveys ask directly.

Researchers should focus on using online and social media data for outcomes where it has an error advantage or a cost advantage, which prohibits surveys from being conducted. This is the case for three reasons.

First, search and social media data can provide insights into the levels of interest and engagement among different online populations. Asking who is responding and when they are doing so, as well as the topics and sentiments expressed, is important. This applies at the individual level as well, potentially allowing for micro-targeting by interest or support. In survey terms, this means for this question the survey error is lower for online and social media data, because the one question we know that online and social media users are answering when they discuss a topic is whether or not they are interested in that topic. Further, researchers can re-weight the data to either a fixed or representative target population using the best practices from non-representative surveys. This provides a more consistent measure to correlate with outcomes such as voting or donations.

Second, online and social media data can be interpreted as a panel response rather than a cross-section, where insights can be gained from studying the shift in individual opinions and behaviors longitudinally. Our detailed data shows individual-level, repeated actions. Using a panel can provide unique insight into how conversations shift over the course of an event. Panels are prohibitively expensive in most traditional survey environments, restricting most traditional surveys to cross-sections, so a naturally occurring panel survey, no matter how imperfect, is better than no panel at all.

Third, this method can construct this imperfect panel ex-post, where ex-post construction is either extremely error prone or impossible for traditional surveys. For example, in traditional surveys the answer to whom respondents voted for in the last presidential election is highly correlated with the current president’s current approval rating [11]. And, as [12] demonstrates with the Gezi protests in Turkey in 2013–2014, ex-post panel construction from social media data allows researchers to have an imperfect, but useful, dataset to examine sentiment for an event that was too unpredictable to create a survey for ex-ante.

The scale and real-time nature of online and social media promises to expand our range of insights into new questions and domains. This paper provides a unique description of a large sample of the data and a new framework for how to approach this type of data. Our study provides a detailed look at why it is so difficult to even understand the selection into online and social media data and the questions that the discussions implicitly answer, which is required before predicting and controlling for these issues. By approaching these challenges from the context of survey research, we can provide a framework for building on established literature in survey research for dealing with non-representation, panels, etc. The directions for future work illustrate how this data can complement traditional surveys. Our study represents a meaningful step towards capitalizing on the strengths of social media data to provide either real-time low-latency or ex-post constructed, temporally-granular, highly-quantifiable answers to relatively-unexplored questions of interest from varying populations.

## Past Research

Much research has demonstrated the promise of online and social media for use in grass roots political mobilization [13], astroturfing [14], predicting outbreaks of influenza [15], reproducing economic indices [16], crisis response [17], and measuring people’s happiness and mood [18,19]. Many studies have documented the existence of a robust and vibrant political discussion on social media across many countries and elections over the past several years [20,21,22,23,24,25]. As participation in online and social media discussions continues to grow more prevalent, monitoring and analysis of these information sources promises to provide new insights into political events and policies. A new working paper [26] highlights how participants view different elicitation techniques from surveys to social media data and the nature of the data. Already, several commercial services track the popularity of political candidates and political issues in online and social media data. Prominent examples include Google Trends (http://www.google.com/trends/) for search and Topsy (http://topsy.com/) for social media. Others papers [27,28] outline further applications of social media monitoring and user classification, including for political advertising.

Many researchers have reported positive correlations between the volume of social media mentions of political candidates and the results of various elections, from presidential elections around the world to US Congressional and UK Parliamentary races [29,30,3]. Similar research has found positive correlations between the volume-weighted sentiments expressed in social media and electoral results and public opinion polls [31,32,2,33]. Notably, prior to the 2012 US presidential election, [34] predicted state-level election results, based on a demographically re-weighted sentiment analysis of tweets. Arguing the necessity of incorporating information beyond volume and sentiment, [35] presents a more sophisticated model that includes behavioral attributes of users, including follower counts and engagement rates, to predict election results. Here we focus less on predicting event outcomes and more on understanding public opinion.

Despite such reports of success, serious questions have been raised about the usage of online and social media in the current literature [5,6,7], including potential methodological issues in data normalization and modeling. Several attempts to reproduce published techniques in the context of different elections have found weak or insignificant correlations or predictions with electoral outcomes [36,37,38,39]. Complementing social media analyses, [7] examines search query volume, and finds no strong correlation between the number of searches for candidates and election outcomes. Others point to the challenges of predicting electoral outcomes based on data that is easily manipulated through astroturfing and “Google-bombing” campaigns [34,40]. While skeptics of the standard methodology have emerged in the last few years, the standard methodology continues to dominate academic literature and there is no consensus around new methodologies.

Recent research has begun to study more deeply the processes that generate online and social media data, including demographic and behavioral attributes of participants. For example, [41] provides a host of self-reported online behavior with a traditional cross-sectional poll of Americans, while [42] examines variation in revealed preferences across demographic groups using web browsing logs from a large online panel. [43] documents the demographic skew on Twitter along with other studies that focus on inferring unknown demographics and attributes of social media users [44,45,46,47], but do little in joining this information with activity and content. On the other hand, papers studying the implications of the online or social media data for politics have mostly ignored demographics [4,2,3], which shift heavily from election to election [41]. A few papers have begun to consider the user, in addition to the text [48,35]. [5] suggests weighting the conversation by demographics and [49] characterizes and models social media participation by politicians and the electorate during the 2011 Spanish presidential election. [50] notes the difference between the selection issues of those who contribute frequently to social media. Highlighting the importance of understanding the implications of participation behavior, [51] finds significant variation in the relative predictive power of subgroups of Twitter users with different behaviors. In the context of web search, both task-based [52] and intent-based [53] classification schemes have been explored. [54] develops a similar taxonomy for Twitter. Shifting and non-stationary intents have been explored [55], but only in the context of optimizing search engines. We have identified very few papers near the political domain that consider the time-series or panel nature of social media data, such as [56,57].

Eliminating selection bias and non-representativeness is a new direction for surveys and we borrow from that budding research for polling convenience samples. [58] shows how even extremely non-representative, opt-in samples can be used as accurate representation of the opinions of a target population with the proper translation of the raw data into an appropriate indicator. We also exploit the panel nature of our data.

Because of the overhead involved with data collection, most research relies on samples of either search or Twitter data to represent online and social media discussions. Prior work using both query log data and the full Twitter stream have been confined to the information retrieval literature [59,60,61], where the focus has been on optimizing search engine effectiveness. Outside of this community, there are limited examples of research utilizing the full Twitter stream [62], but those are mainly focused on examining the network, not the content. [63] shows that there are meaningful sampling issues between the full Twitter stream and any limited streams from Twitter. Several papers have been written by academics within Google, Microsoft, or Yahoo! that utilize the full census of search over a period of time [64,16] but, none of them, to our knowledge, examine the same methods or outcomes as our paper. There is limited work comparing online and social media data to traditional media sources [65].

## Data

Our study focuses on two large-scale datasets representing online and social media data. Our first dataset consists of search queries submitted to major search engines. Our second dataset consists of the complete set of tweets posted during the period of the 2012 election cycle. On the surface, these two platforms share several similarities. Both record short snippets of online, user-generated text; both are indicative of people’s real-time interests; both can be tied to individuals, presenting a longitudinal record of a person’s interests. However, there is also a key difference in people’s intentions while using these platforms. Social media is intended as a public or semi-public communication with other people, whereas search queries are typically intended for private retrieval and consumption of information. Twitter content is typically longer as well, limited to 140 characters, but usually represents a complete thought, while search queries generally contain only three or fewer words, but usually represents a specific information need.

We use ComScore’s records of searches that contain either the keywords “Obama” and/or “Romney” during the 2012 election cycle. ComScore’s panel is well documented and trusted in academic research with over 14,000 hits on Google Scholar as of January 1, 2014. While most of these citations are for their topline results, we analyzed the individual-level responses that comprise those trusted results. Our analysis focused on the subset of ComScore search queries that covers the top three Internet search engines, Google, Yahoo and Bing. ComScore has more detail on their website: http://www.comscore.com/Products/Audience-Analytics/qSearch. ComScore follows a subset of the population, recruited through probability sampling methods including random digit dialing of the general population, so this data is not a census, but it includes comprehensive coverage for these users across major search engines. ComScore pays these 267,518 panel members who are aware of the logging, but they use a very unobtrusive method of data collection, intended to limit the bias of being tracked. We are able to capture whenever a user in their panel searched for either Obama or Romney from July 1, 2012 through November 6, 2012. While anonymized, the ComScore data includes detailed demographics for each user, which they elicit through a survey on entry into the panel, including gender, geographical location, income, and age.

For our social dataset, we use the Twitter firehose—the complete stream of Twitter messages—to capture all of the English language tweets made between August 1, 2012 and November 6, 2012, inclusively, that mention the words “Obama” and/or “Romney”. For each tweet, we capture the text of the tweet, shared URLs, the date and time when the message was written, and profile information about the author, including their name and self-identified location. As Twitter profiles do not explicitly report demographic information, including gender and location, we infer these attributes; this process is detailed in the Appendix. Shortened URLs embedded in a tweet are resolved to their full URLs at the time the tweet is collected.

This results in a dataset with similar information from two of the most meaningful online and social media sources: search and Twitter. For any given search or tweet we have the following: timestamp, a user id, a user name, gender, geography, text, and whether it includes Obama, Romney, or both candidates. We also record the top co-occurring words in the text for each day. For search we have age and income information for the users. We store the data by mentions of Obama only, Romney only, and both candidates for every hour in the dataset, along with counts for overall activity at each time. Table 1 shows some summary statistics of the conversation about Obama, Romney, or both of them, aggregating over the entire period of our dataset from August 1, 2012 through November 6, 2012.

**Table 1:** Summary statistics of demographics associated with search and Twitter for the 2012 presidential election from August 1, 2012 through November 6, 2012 (Election Day). *Note*: Demographics are counted by engagement, not by respondent, so a person’s demographics are counted as many times as they engage. The gender, income, age of the voting population is from 2012 Current Population Survey (CPS). The CPS has an over 90% response rate from their randomly chosen households meant to represent the 129 million voters. The geographical divisions and support represent the true percentages from the 2012 presidential vote totals, with the support shown as percentage of support for each candidate among the two major party candidates. The geographical divisions correspond to the nine official Census Bureau divisions of the United States. We make one small change to divisions to make them more meaningful for politics, which is to shift DC, DE, and MD from South Atlantic to Mid-Atlantic.

	Twitter	Search	Voters
**Obama**	52%	88%	52%
**Romney**	36%	11%	48%
**Both**	12%	0%	0%
**Male**	64%	53%	46%
**Female**	36%	47%	54%
**New England**	5%	4%	5%
Mid-Atlantic*	20%	22%	15%
**East North Central**	13%	14%	16%
**West North Central**	8%	5%	8%
South-Atlantic*	17%	21%	18%
**East South Central**	5%	4%	6%
**West South Central**	11%	10%	10%
**Mountain**	6%	5%	7%
**Pacific**	15%	14%	14%
**Contain URL?**	40%	----	----
**$0-$15k**	----	15%	6%
**$15k-$25k**	----	11%	6%
**$25k-$40k**	----	12%	13%
**$40k-$60k**	----	15%	16%
**$60k-$75k**	----	23%	13%
**$75k-$100k**	----	12%	15%
**$100k**	----	11%	30%
**0–17**	----	9%	0%
**18–24**	----	28%	9%
**25–34**	----	20%	14%
**35–44**	----	15%	16%
**45–54**	----	14%	20%
**55–64**	----	9%	19%
**65+**	----	6%	22%
**Sample Size**	----	----	54,000 in CPS

Some key trends permeate both platforms. First, users discuss Obama much more often than they discuss Romney, likely because Obama is the sitting president and Romney is the challenger. Obama is also the progressive candidate who captured 62% of the 18–29 two-party vote-share while Romney captured 56% of the 65+ two-party vote-share; so this could also be driven by the younger demographics of online and social media. Second, while this varies by platform, males are overrepresented in election-related online and social media activity when compared to their 46% share of the voting population. Third, the northeastern states, comprised of the Mid-Atlantic (i.e. New York, DC, DE, and MD) and New England states, are overrepresented relative to their share of the voting population. Fourth, the demographics of our search data show politically active users are younger than the average voter, with a different income distribution.

The two mediums do differ slightly in their representativeness. First, in raw counts the engagement by candidate is much closer to the voter percentages in Twitter than in search. Second, on gender, search is closer to a representative split than Twitter.

The demographics of social media users do not represent those of the voting population. While unsurprising, this finding is not yet well documented for political discussion. Further, the observed demographics of politically active users on Twitter differ from previous studies of demographics of general Twitter users. For example, 64% of Twitter users are female compared with 43% of individuals who made at least one political tweet during our time period being female and 36% of tweets about election 2014 being sent by females [41]. This indicates that demographics must be topically conditioned, an important finding for social media surveys in general.

We also know that demographics on social media shift over time. Any relationship between social media users and voting populations is unlikely to be the same in 2016 or 2020 as social media shifts within demographic groups much faster and differently than trends among voters. For example, from 2008 to 2010 the population of users between 18 and 22 on social media decreased from 28% to 16%, while the percent between 50 and 65 more than doubled from 9% to 20% [41]. Thus, since age is heavily correlated with political position, the relationship between raw counts and actual outcomes will likely vary over time. For example, the 2012 exit poll estimates that Obama won 60% of 18–29 year olds and 44% of 65+ year olds. Additional examples are available here: http://elections.nytimes.com/2012/results/president/exit-polls.

## Online and Social Media Data as a Survey

In this section, we will demonstrate that there are significant and fundamental structural discrepancies between how survey data and online search and social media data are collected. We support this claim by describing three phenomenon in our data: (1) online user demographics are not only biased, but shift dramatically around major events, (2) individual-level engagement varies dramatically around major events, and (3) the nature of user activity in online and social media discussions also shifts around major events.

### Difficult to Predict Shifting Demographics

Our first major contribution is to show that engagement by demographics is not only unrepresentative overall, shifting between election cycles as previously noted in critiques such as [41], but also shifting dramatically within cycles. All of the statistics in Table 1 are averaged over the last three months of the election cycle; they are not necessarily representative of any point in the election cycle. There are two types of shifts we consider: periodic shifts, following diurnal and septan cycles common in online data, and event-driven shifts, where the demographics and engagement on online and social media dramatically change during a major event such as a presidential debate or breaking news event.

**Fig 1:**
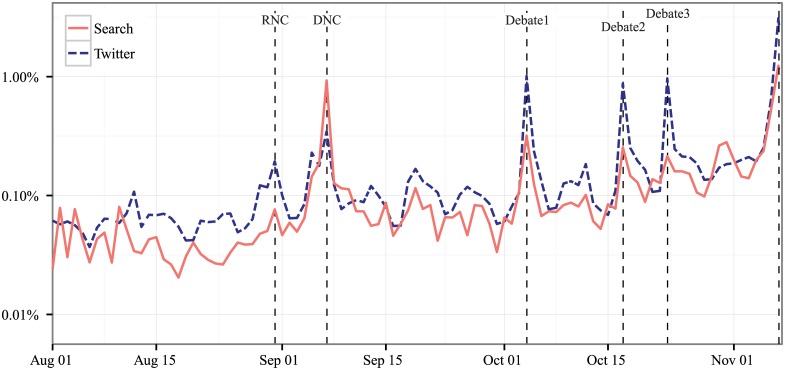
Percent of overall search and Twitter discussion about the presidential candidates from August 1, 2012 through November 6, 2012 (Election Day) on search and Twitter, complied daily. *Note*: Each line combines any text that contains the terms Obama, Romney, or both on any of the two mediums. The charts sum up the total discussion about all three of these categories combined on any given day and divides by the total discussion. The vertical dotted lines in this figure (and almost all other figures) represent the following major events (in order): the Republican National Convention, the Democratic National Convention, and the three presidential debates.

It should not be surprising that activity increases as Election Day approaches. There are also typical daily patterns of discussion. This is similar to traditional surveys and is ultimately not critical to the analysis as long as it is accounted for. However, while traditional surveys hold their samples as consistent as possible, the percentage of online and social media discussion that engages in political discussions shifts dramatically during major events; this movement is much less predictable and thus difficult to correct for ex-ante. Fig 1 shows the percentage of search queries and social media messages that include either a mention of Obama or Romney (or both) during our timeframe (henceforth referred to as *discussion about the presidential candidates*). Notice that the massive jumps during two conventions and the three debates range from a few multiples to 3 orders of magnitude over the normal level of activity depending on the platform and timeframe. For example, in Twitter the day-over-day differences move around 1 order of magnitude for all three debates: 10x, 8x, and 9x respectively, but the search day-over-day differences decline down to just 2x for the last debate after holding steady in the first two debates. The differences are both substantial and difficult to predict with certainty; even the best model is unable to overcome the challenges of continuously evolving and unstable historical data.

**Fig 2:**
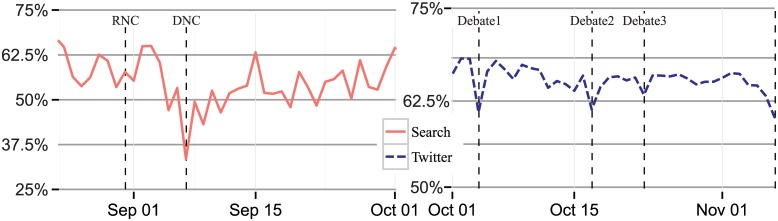
Percent of search and Twitter discussion about the presidential candidates conducted by males from August 15, 2012 through October 1 (search), and from October 1, 2012 through November 6, 2012 (Election Day) (Twitter), compiled daily. *Note*: Each line combines any text that contains the terms Obama, Romney, or both on any of the two mediums. Please note that the y-axis on the left and right are different; the left is twice as large as the right.

Likewise, the overall male to female ratio does not resemble the voting population and it shifts dramatically away from being male-dominated at key moments. Fig 2 charts the percentage of online and social media discussion about politics that males generate. To make the figure most salient, for search we focus on the two major party conventions and for Twitter we focus on the three debates. For completeness, the full plot from August 1 to November 6 is shown in S4 Fig. Not shown on the chart, men actually search more, but women search for politics more often when they search. As a result, women have a similar percent of searches, despite fewer searchers being female. Although this holds steady during the Republican convention, on the final day of the Democratic convention only 33% of searches were done by males, while the average day had a ratio of 55% for males. After that dramatic shift, there was no significant difference in gender representation during the debates. For Twitter, which has a much greater regular imbalance of men to women, the increase in female participation during major events leads to a more representative gender balance. During the three debates the day-over-day difference was 8 percentage points, 5 percentage points, then 3 percentage points respectively. Again the differences in both platforms were both significant and difficult to predict.

**Fig 3:**
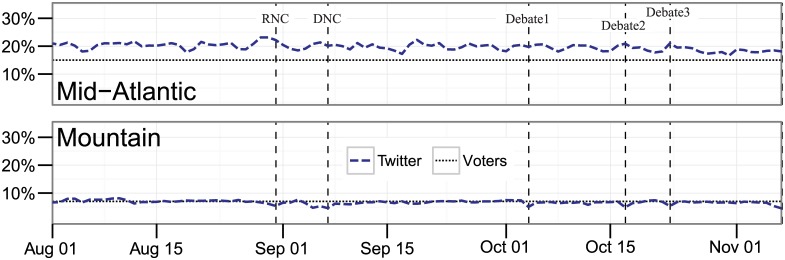
Percent of Twitter discussion about the presidential candidates conducted by geographical division from August 1, 2012 through November 6, 2012 (Election Day), complied daily. *Note*: Each line combines any text that contains the terms Obama, Romney, or both on any of the two mediums.

Similar to gender shifts, there are shifts in the geographical divides during major events as well. Fig 3 charts the percentage of discussion from two geographical divisions during our timeframe for Twitter. For completeness, we have the full plot from August 1 to November 6 for all geographical divisions in S5 Fig. In this salient example of geographical data, the imbalance actually gets worse during major events. The proportion of tweets from the Mid-Atlantic (15% of voters) jumps during the conventions and debates, just as they drop for the Mountain (7% of voters) region. The Mountain region’s drop is sizeable; it averages 6.6% of discussion between August 15 and September 15, 2012, but oscillates between 4.7% and 5.4% during the two conventions. The Mid-Atlantic averages 20.2% during that timeframe and jumps above 23% for the entire Republican convention, but only peaks at 21.3% for the Democratic convention. Again, these are large leaps, but the inconsistency makes it hard to predict and account for these shifts in the data.

What all of these differences and movements in demographics and responses demonstrate is that any index that simply shows the unadjusted response of the population can be misleading. A consumer of an index that does not adjust for changes in the population is not capable of disentangling the meaning of daily or hourly changes. A change in interest levels or sentiment is more likely to reflect shifts in social media engagement (sometimes predictable, sometimes difficult to predict with certainty), rather than any real shifts in interest or sentiment within a consistent group.

### Answering Multiple Times

Traditional surveys limit respondents to a single response. If we wish to consider online and social media discussion as a survey, we must take into account the fact that, unlike in a traditional survey, search and Twitter allow individuals to “respond” multiple times. Here, we move beyond aggregated results and analyze the individual-level responses.

**Fig 4:**
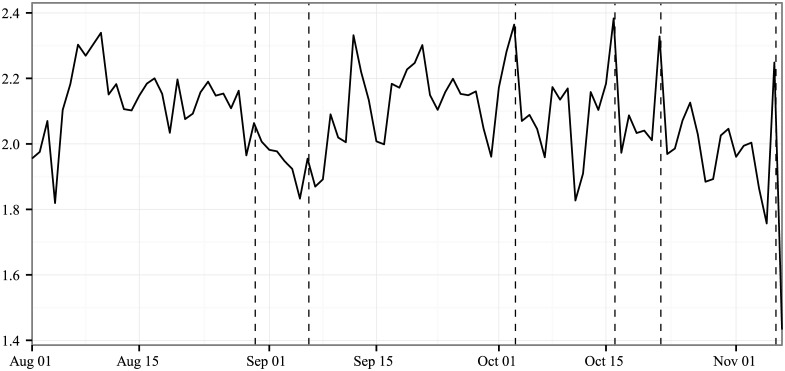
Average number of tweets about the presidential candidates per user from August 1, 2012 through November 6, 2012 (Election Day), complied daily. *Note*: Each line combines any text that contains the terms Obama, Romney, or both on any of the two mediums.

Most discussion is dominated by a small proportion of users: 70% of tweets come from the top 10% of users, with 40% of the discussion from the top 1% of users. Further, this persists over time, with 10% of all tweets generated by the 0.04% of users who tweeted on more than 90% of the days in our sample. Fig 4 shows that the average number of messages per user each day and the typical number of posts per individual rise sharply during the debates. Yet, the variance in individual activity drops on those same days indicating a more equitable distribution of tweets during the key events.

**Fig 5:**
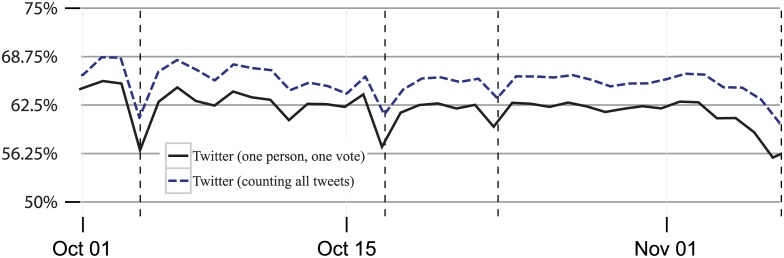
Percent of Twitter discussion about the presidential candidates conducted by males from October 1, 2012 through November 6, 2012 (Election Day), complied daily. *Note*: Each line combines any text that contains the terms Obama, Romney, or both on any of the two mediums. The top line is the same line as the right line from Fig 2.

Since user activity varies substantially across the population, we get a very different view of engagement if we restrict our analysis to one “response” per user per day. Fig 5 repeats Fig 2 and shows the difference in results when we count all tweets compared to counting each user’s contributions only once a day in examining the fraction of election-related activity on Twitter generated by males. We see both an overall shift in the amount of election-related activity from males counting all tweets (an average of 68%) and counting users just once (an average of 63%) as well as different day-to-day movements in these measures. For instance, the drop between the day before the debate and the first debate was 8 percentage points counting all tweets and 9 percentage points counting users only once, and the drop around the second debate was 5 percentage points counting all tweets and 7 percentage points counting users just once. The deviation around the debates appears sharper when users are counted only once.

More surprisingly, we find that if a user engages more than once a day, it is likely not about the same candidate. We show in Table 2, that if a user tweets twice with either a mention of Obama or Romney in the tweet (but not both at the same time) 34% of the time she mentions Obama once and Romney once. If a user tweets three times in a day with either Obama or Romney in the tweet (but not both at the same time) 55% of the time the mix is two for one candidate and one for another candidate. Traditional surveys consistently demonstrate that individuals are more likely to engage in politics if they have strong ideological and partisan position. Likewise, people who tweet about politics are not likely to be ambivalent voters. This provides further evidence that simple counts of mentions are not good indicators of individual-level support [66].

**Table 2:** Percent of users that tweeted just about Obama or Romney, each day, by number of tweets that day for that user that were just about Obama or Romney, from August 1, 2012 through November 6, 2012 (Election Day). *Note*: We drop tweets that include both names, which downwardly biases our results of high levels of “mixed” tweeting. For example, if someone had 4 tweets, with 2 about Obama and Romney and 2 about Obama only, we would put that in the 2 tweet bucket as “All Obama”. For example, if someone had 3 tweets, with 2 about Obama only and 1 about Romney only, that would be under the 3 tweet bucket and “Mixed”.

	Percent All Obama	Percent All Romney	Percent Mixed
**1 Tweet**	54%	46%	0%
**2 Tweets**	34%	31%	34%
**3 Tweets**	23%	22%	55%
**4 Tweets**	27%	16%	66%
**5+ Tweets**	9%	8%	83%

### Shifting Interaction

**Fig 6:**
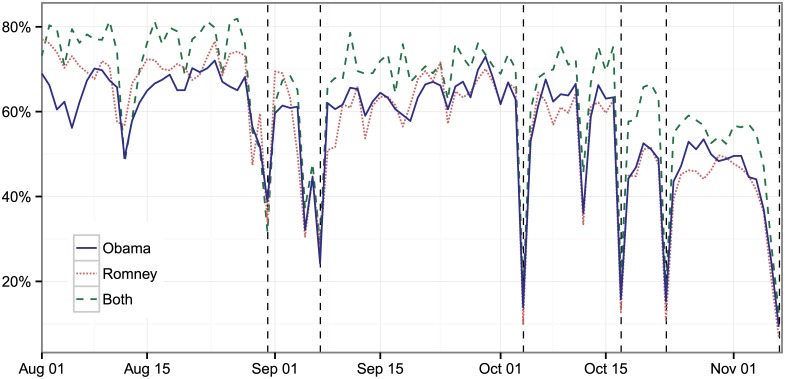
Percent of Twitter discussion about Obama, Romney, and both that contain a URL from August 1, 2012 through November 6, 2012 (Election Day), complied daily. *Note*: Each line shows the percentage of tweets with a URL about all three of the categories separately on any given day.

The nature of user interaction shifts around major events. For example, an election-related tweet containing a URL is likely to be informational in nature, linking to media around a candidate, whereas tweets expressing opinions and support may contain fewer links to additional content. Fig 6 charts the percentage of election-related tweets with a URL, showing dramatic daily shifts. Overall, 62% of election-related tweets the day before the first debate contained a URL and only 12% of tweets the day of the first debate did. The shift is even more pronounced for discussions involving Obama. The day before the first debate Obama or Romney tweets were about equally likely to contain a URL, but the day of the debate a tweet mentioning Obama was 1.4 times more likely to contain a URL than one mentioning Romney.

**Fig 7:**
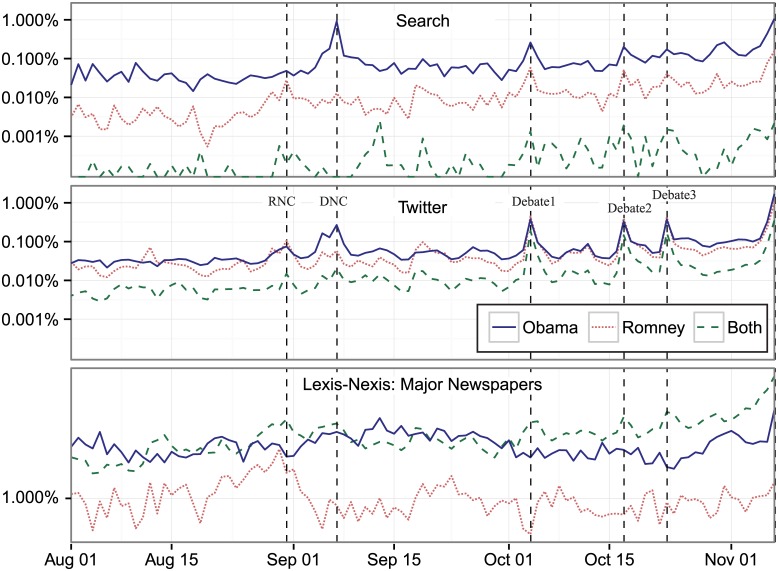
Percent of overall search, Twitter, and print media discussion about the Obama, Romney, and both from August 1, 2012 through November 6, 2012 (Election Day), complied daily. *Note*: Each line sums up the total discussion about all three of the categories separately on any given day and divides by the total discussion.

These changes are not uniform across different platforms, which answer different questions about interest at different times. Fig 1 shows how the discussion levels for Obama, Romney, and both move during the election cycle. We compare this engagement with the mainstream media and break it down by each of the three topics. Fig 7 shows the progression of the discussion about Obama, Romney, or both in search, Twitter, and mainstream media. As a proxy for the mainstream media we use the number of indexed stories by Lexis-Nexis, which covers an array of general news sources. First, notice that Obama is always searched for the most; people have an interest in learning about the sitting president, although Romney does top Obama at certain points in time. Second, the mainstream media slowly builds up coverage around major events and then slowly winds them down, while online and social media hit the event with a more sudden burst. For example, while there are sharp differences in the discussion level about Obama, Romney, and both on the three debate days in search and Twitter, the discussion is relatively flat in the major newspapers. Third, there is complex relationship between these trends, but for overall interest, mainstream media leads online and social media. For example, a one-day lag of overall mainstream media discussion level is statistically significant in predicting Twitter’s level of discussion, while there is no significance in the other direction. Online and social media data provide a different interest index than that of the mainstream media.

**Fig 8:**
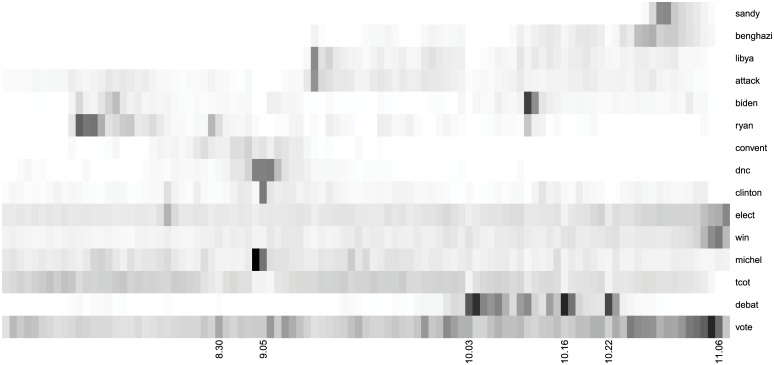
Top terms co-occurring with “Obama” on Twitter, from August 1, 2012 through November 6, 2012 (Election Day), complied daily. *Note*: Term rows are arranged by time series clustering.

We also examine the terms that co-occur with candidate mentions to gain further insight into the nature of political discussion via online and social media data. While the earlier figures focus mostly on engagement, co-occurrence of words is closer to domain-specific sentiment. Fig 8 shows a selection of the top terms mentioned alongside Obama on Twitter over time, each day, where darker cells indicate higher interest (the corresponding figure for search for Obama is S1 Fig, Twitter for Romney is S2 Fig, and search for Romney is S3 Fig). We start by including all terms occurring more than 100,000 times in tweets mentioning Obama or Romney, after removing stop words. For each term, we normalize daily values by the total count for that term over all days in our experiment. We then cluster these terms according to Euclidean distance using the heatmap.plus R package (http://cran.r-project.org/web/packages/heatmap.plus/index.html). The final figures, Fig 8 and S1,S2 and S3 Figs, illustrate a selection of politically salient terms. (Additional terms are omitted due to space constraints.) First, this provides further verification of how the discussion changes between general interest terms to trending terms around specific events. Second, important for the campaigns, charts like Fig 8 can provide a quick and clear indicator of how their messaging is received by the different populations. We can spot the evolution of terms on both platforms that the campaigns are either promoting or avoiding. For instance, this illustrates how the attacks on the U.S. embassy in Benghazi, Libya shift from being “attack” and “Libya” into “Benghazi”. We can focus on major events and see what people are discussing. During the Democratic Convention, searches were dominated by queries about Obama’s family members: Michelle, Sasha, and Malia, but tweets focused more on policy terms and Bill Clinton. We can compare across candidates and see that while Hurricane Sandy and related terms dominated the discussion around Obama in the days before the election, Romney was battered with searches regarding false claims in his advertisement about Jeep. Third, domain experts know Jeep as a negative term for Romney and Benghazi as a negative term for Obama (while Libya was a neutral term); this type of chart is a powerful sentiment indicator for domain experts. We refer to this as domain-specific sentiment, because the terms shift over time and candidates, making it difficult to build a lexicon. Benghazi drifted into a negative term for Hillary Clinton while Jeep would now be seen as neutral term.

## Discussion

This paper utilizes large corpora of search and Twitter data during the 2012 election cycle to provide a multi-platform view of online and social media data. We find that if search and Twitter data are to be treated as a survey, they would follow a very peculiar methodology: participants are a time-varying, demographically-biased sample of the population who effectively answer different “survey” questions to questions of their choice with varying frequency. Exactly how these data should be utilized to complement existing polls and surveys is an open question. We do not propose to have all of the answers, but by understanding the data we hope to direct future research and methodology towards this goal.

### Levels of Interest, Engagement and Reweighting

The main results of this paper already show that online and media data can provide insights into the levels of interest and engagement among different population demographics. Practitioners and academics should not ignore these results as there are certainly situations where they are valuable outcomes for stakeholders. However, we also propose continuously re-weighting the data to a specified target population; either a consistent representation of online or social media users or the voting population. We expect this new direction to unlock the potential of online and social media data.

There are two key reasons to re-weight data to reflect the composition of known, stable populations: to provide meaningful aggregated comparisons and create consistent correlations with key outcomes. For example, women are underrepresented in the raw volume of tweets, but tweet more often about politics than men. Thus, the average level of conversation increases when we weight by women representatively. If we want to provide an account of the general population, then it is key to re-weight by gender, age, geography, individual, etc. Since demographics of active users shift so much over time (e.g., geography and age) it is also key to provide a stable demographic sample if correlations to outcomes the campaigns care about are going to hold over time and elections. In the literature survey at start of this paper, we mention predicting influenza with search data, which is one of the most popularized examples of the utility of online data. That said, recent work [67] shows that that relationship failed soon after the initial papers were published---correlating topline results with outcomes is fragile when the underlying input data is in constant flux. [68] and others attempt to solve this particular problem for influenza with a more nuanced approach to the data, but this lacks of out-of-sample robustness is still dominant in both academic research and practice. The more granularity we can exploit in the input data, the more stable the answers derived from it will be.

We propose using the same methods for re-weighting the data as the new methods being tested for non-representative surveys, but surveys are ahead in practical use of this methodology. First, surveys have the ability to directly ask for, rather than infer, demographics. As noted in the Appendix, there is still substantial error in inferring the demographics of users online, which influences the accuracy of any estimates researchers derive with those imputed demographics. However, the processes for matching demographics are improving rapidly as users provide more profile information, more datasets are linked between individuals, and methodology for analytics improves. Second, there is still uncertainty in the accepted methodology as traditional re-weighting approaches championed by the polling community for transparency and theoretical foundation (e.g., [69]) are compared with more aggressive Bayesian multi-level regression and post-stratification championed by papers like [58]. There is the possibility that more aggressive approaches could overcorrect or cause other issues. Finally, there is no reason to confine re-weighting of social media data to traditional demographics, as a host of psychological or network demographics could prove to create more accurate corrections for social media data.

### Online Data as a Panel Response

**Fig 9:**
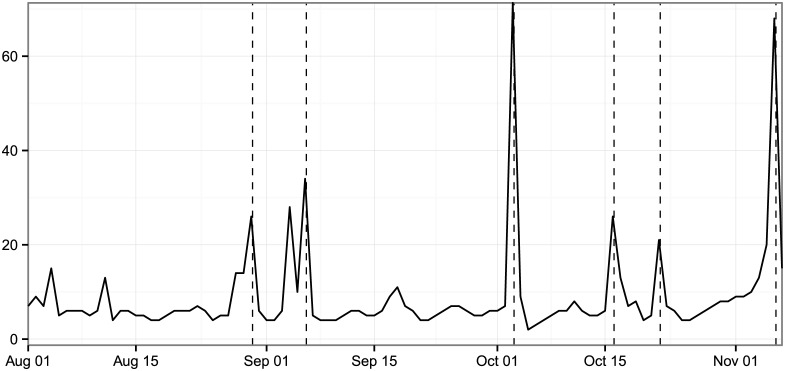
Median days since the last election-related tweet by the same person for any given tweet on that day. *Note*: October 3 is actually infinity in that the median tweet was created by a person who did not tweet about the election in our sample.

Another methodology for utilizing the data is to treat online and social media as a panel, where we can track the opinions of a set of users over time. For instance, in Fig 9 we show the median days since the last election-related tweet by the same person for any tweet on a given day. For most days in the sample this distance is within a week—the median is 6 days. This means that if one picks a randomly selected tweet from a random user on a typical day, that user’s last election-related post occurred less than a week earlier. However, we see a drastic shift in this quantity around conventions and debates. For instance, more than half of the activity on the October 3^rd^ debate came from individuals who had not previously posted about the election. These are likely to be a very different subset of the population than regularly engaged users. At the same time, this repeated usage shows the promise of capturing when and how conversations change, not just between users, but within users.

Further, these panels can be constructed ex-post to capture treated and control users of different events. This is especially relevant if the event was surprising or affected a specific demographic. Researchers can randomly select users and follow them through an event, which is standard in survey research, rather than select users because they engaged with a topic, which is standard in this type of research. In the theory of survey design, it is almost always better to choose users randomly and observe how they shift over time, but the depth is limited. Fig 1 shows only 0.1% to 1.0% of tweets or searches talked about the election in the weeks prior to Election Day. Panels of 100,000 or even 1,000,000 users, especially broken down by demographics, may generate relatively little conversation during any particular day.

We do not go into detail on sentiment in this paper for two key reasons. First, the same selection issues affecting counts affects per-tweet sentiment, thus we see counting as more fundamental to describing the pertinent challenges and sentiment as a secondary issue to explore in later work. The best sentiment models still have a relatively low recall, so they only cover a fraction of the total discussion. Second, we view co-occurring words as a crude, domain-specific sentiment for domain experts. Sentiment models on Twitter data differ from public opinion on major topics, but the current state-of-art cannot clarify whether this is due to a true difference in sentiment or simply an artifact of the models themselves [70]. Future work is already underway in using co-occurring words to create continuously updating domain-specific sentiment.

These results should be interesting for campaigns or observers who are invested in following how ideas propagate through the population with quantitative precision and low latency. We are not pursuing matching the ground truth of an election outcome or current level of support here, but instead a new metric that could be used to study the impact of the billions of dollars campaigns spend each cycle on the interest levels and discussions during the campaign. Campaigns adjust their messages daily (or even hourly) and our concepts of low latency and quantitative indicators of interest can help determine the effectiveness of those messages within detailed demographic groups. Further, the methods outlined in the paper will also help target the individuals as they are either followed directly or as part of a demographic group.

## Appendix

We use the tweet author’s first name to impute gender, based on gender distributions gathered from annual social security reports of births from the years 1880 to 2012. This dataset provides us with gender probabilities for 91,320 names, with almost all names clearly indicating one gender or the other (91.7% of names are single gender with 99.0% probability). We leave the values as the percentage likelihood of being male rather than making a binary choice on gender. This dataset provides 58% coverage over Twitter user profiles and 54% coverage over messages. Twitter user names that are not covered by our technique include pseudonyms without first names, organizational names, and foreign names too uncommon in the US to be included in the list of SSN births; this is a common procedure [71].

We infer a tweet author’s coarse-grained geographic location from their self-declared user profile [72]. While people often self-declare easily interpretable geographic locations, many use colloquial names (“deep south”), acronyms (“DMV” for DC-Maryland-Virginia) and terms that are unrelated to locations (“none of your business”, “the universe”). To learn the physical locations of these self-declared location names, we collect 1 month of geo-located tweets---separate from our election-related tweets--- where individual tweets have been tagged with a specific latitude and longitude, in addition to the users’ self-declared location names. From this, we are able to learn a mapping from self-declared location names to latitude-longitude ranges. We extend this learned mapping with a list of US cities. For our analyses in this paper, we translate user locations to US regions and divisions. International, ambiguous, and unrecognized locations are labeled separately. The location is determined in 48% of profiles and that accounts for 56% of tweets; we ignore the 21% of profiles that are identified to be international, but they comprise just 13% of geography-identified tweets. [73] provides a detailed look at where geographical location from social media can have even more errors than generally assumed in the literature.

## Supporting Information

S1 Fig Top terms co-occurring with “Obama” on search, from August 1, 2012 through November 6, 2012 (Election Day), compiled daily. *Note*: Term rows are arranged by time series clustering.
(EPS)

S2 Fig Top terms co-occurring with “Romney” on Twitter, from August 1, 2012 through November 6, 2012 (Election Day), compiled daily. *Note*: Term rows are arranged by time series clustering.
(EPS)

S3 Fig Top terms co-occurring with “Romney” on search, from August 1, 2012 through November 6, 2012 (Election Day), compiled daily. *Note*: Term rows are arranged by time series clustering.
(EPS)

S4 Fig Percent of search and Twitter discussion about the presidential candidates conducted by males from August 1, 2012 through November 6, 2012 (Election Day), compiled daily. *Note*: Each line combines any text that has Obama, Romney, or both on any of the two mediums.
(EPS)

S5 Fig Percent of search and Twitter discussion about the presidential candidates conducted by geographical division from August 1, 2012 through November 6, 2012 (Election Day), compiled daily. *Note*: Each line combines any text that contains the terms Obama, Romney, or both on any of the two mediums.
(EPS)
